# Institutional readiness to provide critical care to patients with viral hemorrhagic fever (VHF) in the United States after the COVID-19 pandemic

**DOI:** 10.1017/ash.2025.10167

**Published:** 2025-10-13

**Authors:** Madeline A. DiLorenzo, Anthony LoPiccolo, Wael ElRayes, Jocelyn J. Herstein, Radu Postelnicu, Angela Vasa, Vikram Mukherjee

**Affiliations:** 1 Division of Infectious Diseases, Department of Medicine, New York University Langone Health, New York, NY, USA; 2 Division of Infectious Diseases, Department of Medicine, New York City Health + Hospitals/Bellevue, New York, NY, USA; 3 Special Pathogens Program, NYC Health + Hospitals/Bellevue, New York, NY, USA; 4 Division of Pulmonary, Sleep and Critical Care Medicine, Department of Medicine, New York University Grossman School of Medicine, New York, NY, USA; 5 Division of Pulmonary, Sleep and Critical Care Medicine, Department of Medicine, NYC Health + Hospitals/Bellevue, New York, NY, USA; 6 College of Public Health, University of Nebraska Medical Center, Omaha, NE, USA; 7 Global Center for Health Security, University of Nebraska Medical Center, Omaha, NE, USA; 8 Center for Global Health and Development, University of Nebraska Medical Center, Omaha, NE, USA; 9 Nebraska Medicine, Omaha, NE, USA

## Abstract

**Objective:**

A follow-up survey of Special Pathogen Treatment Centers (SPTCs) was conducted to assess their readiness to provide critical care interventions to confirmed and suspected viral hemorrhagic fever (VHF) patients after the COVID-19 pandemic.

**Methods:**

An electronic survey with 54 multiple-choice and free-response questions assessing VHF critical care capabilities was sent to 74 US SPTCs in April 2023.

**Results:**

Fourteen SPTCs (19%) completed the survey. Most respondents were prepared to provide intubation/mechanical ventilation (79%), pharmacologic cardioversion (79%), renal replacement therapy (71%), and defibrillation (71%) to suspected and confirmed VHF patients. Few were ready to provide cricothyrotomy (36%), extracorporeal membranous oxygenation (ECMO) (29%), or code status (14%). Factors impacting institutions’ ability to provide critical care to a VHF patient included staff safety (71%) and clinical futility (50%). Less than half (36%) reported that the COVID-19 pandemic positively affected their facility’s ability to care for VHF patients, while 21% indicated the pandemic prompted their facility to be better prepared to care for a VHF patient.

**Conclusions:**

Most responding SPTCs reported capability for critical care interventions, although fewer had policies governing code status, ECMO, and cricothyrotomy. Institutions were less prepared to manage a VHF patient after the COVID-19 pandemic, highlighting challenges such as staff turnover and less support for training and equipment maintenance. Given that special pathogen outbreaks continue to occur globally, governments and healthcare institutions should institute measures to recruit, support, and retain staff to ensure critical care readiness for special pathogen patients.

## Introduction

During the 2014–2016 West Africa Ebola virus disease (EVD) epidemic, the US government designated certain US healthcare institutions as Special Pathogen Treatment Centers (SPTCs).^1^ There were 56 SPTCs when they were established in 2016.^2^ At the same time, the Assistant Secretary for Preparedness and Response, later renamed to Administration for Strategic Preparedness and Response (ASPR), established the National Ebola Training and Education Center, now renamed the National Emerging Special Pathogens Training and Education Center (NETEC), a consortium of 3 healthcare institutions that treated patients with EVD in 2014: Emory University, the University of Nebraska Medical Center/Nebraska Medicine, and New York City Health and Hospitals/Bellevue Center. NETEC’s mission is to prepare US healthcare facilities to care for patients with special pathogens, including viral hemorrhagic fevers (VHF) such as EVD, Lassa fever, and Marburg virus disease (MVD). However, the number of SPTCs has fluctuated over time, with several facilities recently decommissioning their SPTC capabilities due to inadequate funding. ^3,4^

Since NETEC was established, there have been multiple VHF outbreaks. In 2024, Rwanda experienced its largest MVD outbreak to date. In 2025, there have been outbreaks of MVD in Tanzania, Ebola Sudanvirus in Uganda, Lassa fever in Nigeria, Crimean–Congo hemorrhagic fever in Iraq, and Nipah virus in India.^5–11^ The need to support institutional readiness for VHF within the United States was further reinforced after a patient returning from West Africa passed away from Lassa fever in Iowa in October 2024.^12^

Given the lack of medical countermeasures for VHFs, the primary treatment for these illnesses with very high baseline mortality is supportive critical care.^13^ This was highlighted during the EVD outbreak in 2014, where most patients treated in the United States and Europe survived, likely because they had access to timely critical and supportive care that is not always available in resource-constrained settings.^14,15^ Given this, NETEC has developed numerous resources to support critical care delivery for patients with VHF. However, there remains a wide range of institutional comfort and experience with providing critical care interventions to these patients. Furthermore, there is a lack of national consensus on which interventions should be offered and when.

A 2020 prepandemic survey of SPTCs found that most were prepared to provide intubation, chest compression, and renal replacement therapy (RRT) to suspected or confirmed VHF cases but were not ready to provide interventions such as extracorporeal membranous oxygenation (ECMO) and cricothyrotomy.^16^ Conducted in the early months of the COVID-19 pandemic, the survey provided a snapshot of SPTCs’ VHF critical care capabilities before the pandemic caused widespread impacts to the healthcare system, including healthcare worker burnout and attrition.^17^ To determine if the strain from the pandemic affected SPTC capabilities to provide critical care interventions to VHF patients, the authors, several of whom contributed to the 2020 survey, developed a follow-up survey structured to mirror the 2020 survey. This study reassesses SPTC readiness for critical care interventions and examines the COVID-19 pandemic’s impact on the training, staffing, and VHF readiness strategies of facilities to provide care to a suspected or confirmed VHF case.

## Methods

### Study instrument

A 54-item survey was prepared using RedCap (Appendix A). The survey asked participants about institutional capabilities in 9 critical care areas: (1) RRT, (2) endotracheal intubation and mechanical ventilation, (3) ECMO, (4) chest compressions, (5) pharmacologic cardioversion, (6) electrical cardioversion, (7) defibrillation, (8) cricothyrotomy, and (9) code status. For each of these areas, participants were asked to respond to a “yes/no” question on whether their institution had a policy and to summarize their policy if one existed. They were also asked to indicate if (a) they had prior experience providing a given critical care intervention to a suspected or confirmed VHF case, (b) whether they were currently ready to provide that intervention to either a suspected and/or a confirmed VHF case and (c) if their institutional policy stated that a given intervention would not be offered to a suspected and/or a confirmed VHF case.

Participants were also asked to rate, on a 5-point Likert scale with 1 being “*Does not limit care*” and 5 being “*Limits Care,*” how much the following 5 factors influenced their ability to provide critical care to a suspected or a confirmed VHF case: (1) staff safety, (2) lack of appropriate technology, (3) lack of guidelines on how to provide care, (4) clinical futility, and (5) limitations of the physical building or ward. They were also asked about how their strategies around staffing, technology, and training for VHF preparedness had changed since the COVID-19 pandemic, and whether their institution felt more or less prepared to care for both confirmed and suspected VHF cases since December 2019.

### Inclusion criteria

The survey was distributed by email to representatives from 74 different institutions that had been identified as SPTCs either through designation as a treatment center in 2014 or through their affiliation with NETEC.^2,18^ Participants could take part in the study if they were a clinician involved in an active or previously designated SPTC program and were knowledgeable about their program’s VHF treatment protocols. Each institution only submitted one response.

### IRB approval and participant consent

This study was approved by the New York University Langone Health Institutional Review Board (H-38993). Consent was obtained through an introductory email that described the study goals. Participation was voluntary. If a participant chose to start the survey, they were considered to have provided consent. All responses were de-identified upon receipt.

### Study period

The survey was sent on April 5, 2023, with 2 email reminders sent to identified contacts at 1-month intervals. Data collection concluded on July 18, 2023. We calculated frequencies and percentages for ordinal and categorical data pertaining to the participating centers’ characteristics and policies. For qualitative data about the participating centers’ critical care policies, perceived challenges, and innovations used to provide care, we conducted a thematic analysis to identify recurring patterns and themes. For the Likert scale analysis, responses of 4 and 5 were considered to indicate that the factor limited the facility’s ability to provide care.

## Results

### Respondent characteristics

Fourteen institutions (19%) completed the survey. One (7%) no longer considered itself an active SPTC, although they indicated they maintain the capability to care for a suspected or confirmed VHF case. All Health and Human Services (HHS) regions except Region 8 were represented with the most (29%, n = 4) responses coming from HHS Region 3 (Table 1). Most (57%, n = 8) individuals who completed the survey were physicians working in critical care, emergency medicine, or infectious diseases. The other respondents held roles in nursing, hospital epidemiology, and institutional administration.

**Table 1.** Descriptive data on survey respondents (eg, clinical role, region, etc.)

Respondent demographics n=14	Respondents (%)
**HHS region**	
1	2 (14.3)
2	1 (7.1)
3	4 (28.6)
4	2 (14.3)
5	1 (7.1)
6	1 (7.1)
7	1 (7.1)
8	0 (0.0)
9	1 (7.1)
10	1 (7.1)
**Role within institution**	
Administration	1 (7.1)
Nurse	3 (21.4)
Physician	9 (64.3)
Other healthcare worker	1 (7.1)
**Role within SPTC**	
Emergency management	2 (14.3)
Medical director	8 (57.1)
Administration	2 (14.3)
Clinician	2 (14.3)
**Department**	
Critical care	6 (42.9)
Emergency medicine	2 (14.3)
Infectious disease	2 (14.3)
Emergency management	2 (14.3)
Other	2 (14.3)
**Median year SPTC founded (IQR Q1–Q3, N=12)**	2015 (2014–2016)
**Active SPTC**	13 (92.9)
**Has taken care of a suspected VHF**	6 (42.9)
**Has taken care of a confirmed VHF**	0 (0)

Demographic information of the respondents and the institution in which they work. Note. HHS, Health and Human Services; SPTC, Special Pathogen Treatment Center; IQR, interquartile range; VHF, viral hemorrhagic fever.

### Policy scope, patient experience, and institutional readiness

Most responding SPTCs were ready to provide intubation/mechanical ventilation (79%, n = 11), pharmacologic cardioversion (79%, n = 11), RRT (71%, n = 10), and defibrillation (71%, n = 10) to a confirmed VHF case (Table 2). Few were prepared to provide cricothyrotomy (36%) and ECMO (29%) to confirmed VHF patients, and fewer had specific policies around code status (14%). Institutional policy details from those that provided them (n = 10) are in Table 3.

**Table 2.** Readiness to provide critical care interventions for confirmed adult VHF patients

Critical care capability	SPTC n=14 (%)
Renal replacement therapy	10 (71.4)
Intubation/mechanical ventilation	11 (78.6)
Extracorporeal membrane oxygenation	4 (28.6)
Chest compressions	9 (64.3)
Pharmacologic cardioversion	11 (78.6)
Electrical cardioversion	9 (64.3)
Defibrillation	10 (71.4)
Cricothyrotomy	5 (35.7)
Code status	2 (14.3)

Critical care capabilities that respondents were ready to provide to a confirmed adult VHF patient. Note. VHF, viral hemorrhagic fever.

**Table 3.** Critical care policy details for suspected and confirmed VHF cases

Policy area	Institutions with policy (n, %)	Offered to all patients	Case-by-case	Would not offer	Respondent notes and comments
RRT	7 (50.0)	4	3	0	- Physician and nursing leadership consensus - One institution would only offer only in cases of single organ failure - Line placement done in biocontainment unit
Intubation/ mechanical ventilation	10 (71.4)	7	3	0	- Just-in-time training for relevant staff - Appropriate PPE available - Sufficient staffing (eg, multiple providers in room) - Assigning a designated ventilator(s) to special pathogen unit
ECMO	5 (35.7)	0	1	4	None
Chest compressions	10 (71.4)	3	5	2	- Cause of arrest was easily reversible - One institution would offer chest compressions to a suspected, but not confirmed VHF patient - One institution noted that their state law does not allow for patients to be DNR without their consent, so in that case, CPR would be performed if the patient could not confirm they wanted to be DNR. This applied to all interventions related to CPR and code status. - Some institutions used a Lucas device operated by trained in-room RN and MD
Pharmacologic conversion	9 (64.3)	6	3	0	- Many institutions had similar parameters to what they had for chest compressions
Electrical cardioversion	9 (64.3)	5	3	1	None
Defibrillation	9 (64.3)	5	3	1	None
Cricothyrotomy	3 (21.4)	0	1	2	None
Code status	2 (14.3)	0	2	0	- One policy would make patients DNR for chest compressions and emergent intubation (unless capable staff already in PPE) - One would perform all elements of CPR because their state law prohibits making patients DNR without their consent

This table displays the institutions that have hospital policies surrounding critical care interventions for VHF patients. Note. VHF, viral hemorrhagic fever; RRT, renal replacement therapy; PPE, personal protective equipment; ECMO, extracorporeal membrane oxygenation; DNR, do not resuscitate; CPR, cardiopulmonary resuscitation; RN, registered nurse; MD, doctor of medicine.

### Factors influencing the provision of critical care

When determining which critical care interventions to provide to a suspected case, most (71%) institutions named staff safety, and half (50%) cited clinical futility as a factor influencing the decision (Table 4). Few institutions cited lack of appropriate technology (36%), lack of care guidelines (36%), and building/ward limitations (21%) as factors. The response rates for each factor were the same for both suspected and confirmed VHF cases, except for clinical futility, where the proportion of participants citing this as a concern increased from 50% to 64%, respectively.

**Table 4.** Factors impacting clinical care of a VHF patient

Potential barriers	Suspected patient	Confirmed patient
Staff safety	71%	71%
Lack of appropriate technology	36%	36%
Lack of care guidelines	36%	36%
Clinical futility	50%	64%
Building/ward limitations	21%	21%

Barriers that impact the ability of survey respondents to provide critical care to a suspected and a confirmed patient. Note. VHF, viral hemorrhagic fever.

### Strategies that have changed during the COVID-19 pandemic

Six (43%) institutions cited no changes to their strategy for providing critical care to VHF patients due to the COVID-19 pandemic. Among the remaining 8 institutions, all cited changes to their training strategy, 4 cited changes to their staffing strategies, 2 changed their philosophy on resuscitating a suspected or confirmed VHF patient, and 1 changed its technology strategy.

Less than half (36%) of institutions stated that the COVID-19 pandemic positively affected their ability to care for a suspected or confirmed VHF patient. Factors contributing to this included increased awareness by hospital leadership on the importance of maintaining a diverse, well-trained special pathogens team, better understanding of staffing challenges and the impact of long-term unit activation, better awareness of personal protective equipment (PPE) utilization and donning/doffing skills, and the ability to perform surge maneuvers. Another 36% stated that the COVID-19 pandemic negatively affected their ability to provide critical care to VHF patients, citing staff attrition, PPE training burnout, and inability to maintain regular training sessions as contributing factors. The other 28% of institutions did not feel the COVID-19 pandemic impacted their institutions’ ability to care for VHF patients.

As far as preparedness to care for a suspected or confirmed VHF case, 21% of institutions felt more prepared due to increased real-world experience and a stronger culture around the importance of PPE use. In contrast, 43% of institutions felt less prepared to care for VHF patients, citing less trained staff, difficulty providing specialized VHF PPE training to staff, staff turnover, and infrequent equipment testing as factors. The remaining 36% of institutions felt there was little to no change in their preparedness since prepandemic.

## Discussion

The US network of SPTCs is crucial to maintaining the nation’s special pathogen preparedness capabilities. This survey included fewer institutions than the one conducted in 2020, but several SPTCs have been decommissioned since the original survey was conducted.^4^ As in 2020, most institutions felt comfortable providing RRT, intubation/mechanical ventilation, chest compressions, pharmacologic conversion, and electrical cardioversion to patients with VHF.^16^ Providers continue to have less comfort and experience providing ECMO and cricothyrotomy to VHF patients. The data we found in this and our prior survey are similar to that found in a recent NETEC survey of 10 federally funded Regional Emerging Special Pathogen Treatment Centers (RESPTCs).^1^ These RESPTCs were surveyed on only 3 of the policy areas queried in this survey: RRT, intubation/mechanical ventilation, and ECMO. All were able to provide RRT and intubation/mechanical ventilation to patients, while only 40% were ready to provide ECMO.

A review of the literature highlighted the benefits of a special pathogens program during the COVID-19 response. In a 2020 survey of 20 hospitals that had an onsite consultation with NETEC, 95% of institutions reported PPE shortages, but 80% were able to use their special pathogens program’s PPE to support their COVID-19 response, and 63% used their biocontainment unit to provide education to frontline staff.^19^ A similar 2021 survey of SPTCs found that trained special pathogen response staff, shared developed protocols, and donated supplies improved their COVID-19 response.^4^

However, despite these prior studies’ findings, only 36% of participants felt that COVID-19 positively impacted their ability to care for VHF patients, and only 21% felt more prepared. Several factors may explain this discrepancy in reported preparedness after the COVID-19 pandemic. First, the expertise and training required to care for a COVID-19 patient compared to a VHF patient are different, with the latter requiring more complex PPE and donning and doffing procedures. Second, the COVID-19 pandemic has led to significant burnout among healthcare workers. Although it is challenging to precisely quantify, higher rates of physicians, nurses, nurse practitioners, and physician assistants have left healthcare in the years since the pandemic started than in the years before it and are being replaced by younger staff with less experience and expertise.^20,21^ This burnout has been linked to concerns about staff safety, which in this study was cited as the most important factor when determining whether to provide care to patients with suspected or confirmed VHF.^22^ Furthermore, although our survey focused on the United States, global high-level isolation units are also experiencing significant staffing shortages, which will affect the management of high-consequence pathogens globally.^23^

Although the COVID-19 pandemic may have left some institutions feeling less prepared to care for VHF patients than previously reported, there are opportunities to support healthcare workers to safely provide life-saving critical care to VHF patients. Consistent training on enhanced infection prevention and control practices—particularly in practical exercises such as simulated patient drills—would help staff develop and retain the confidence to care for VHF patients.^24,25^ Improved communication between SPTCs and regional RESPTC(s), NETEC, ASPR, and the Centers for Disease Control and Prevention could help ensure that providers are up to date on care guidelines and are able to share lessons learned. RESPTCs in particular are tasked with supporting and maintaining VHF preparedness efforts for their region through resource sharing during both peacetime and outbreaks. For example, project ECHO, a collaborative learning platform, was used during the COVID-19 pandemic to promote real-time peer-to-peer learning.^26^ Similarly, RESPTCs and NETEC regularly host webinars to share information on special pathogen threats and best practices.^27–29^ By improving communication between SPTCs, providers in different locations will be able to quickly learn from other institutions and tailor innovative strategies to their own setting.

As in our 2020 survey, clinical futility was still seen as a major barrier to providing clinical care to VHF patients, particularly for confirmed cases. Although there are certainly cases of VHF where providing critical care interventions would be futile and would put staff at risk, the US experience during the EVD outbreak in 2014 highlights that when patients were able to get to well-resourced institutions that could safely provide medical care—including critical care interventions such as intubation and dialysis—their survival rate was much higher than that of patients in resource-limited settings.^30,31^ This highlights the importance of dispelling the notion that the provision of critical care to patients with VHF is futile. Enhancing the critical care capabilities of SPTCs by providing ongoing support for dedicated staff, training, and equipment will ensure that the necessary care these patients require can be safely provided and positively impact survival after VHF infection.

This study had several limitations. First, only 19% of those who received a recruitment email responded to the survey, making it difficult to draw reliable conclusions. Second, most respondents were physicians and may have a biased perception of the impact that the COVID-19 pandemic had on their program compared to someone in an administrative or leadership role. At the same time, physicians and nurses in critical care are often the most knowledgeable on their institutions’ capabilities to provide care to a VHF patient, so this may mitigate potential bias.

## Conclusion

Our study found that institutions are prepared to offer many critical care interventions to patients with VHF. However, they could benefit from additional support to train and retain staff and to develop the expertise to provide other life-saving critical care interventions to VHF patients, namely ECMO. NETEC and RESPTCs strive to provide this support by creating a community to strengthen SPTCs, and this effort was further cemented when Congress directed NETEC to be the coordinating body of the National Special Pathogens System of Care (NSPS) in 2024.^32^ NSPS’s goal is to institutionalize special pathogen capabilities and practices across all levels of the US healthcare system. Given that we continue to see outbreaks of VHFs and other high-consequence pathogens (mpox) globally, institutional and governmental support for SPTCs, such as the development of the NSPS network, will be crucial going forward.

## Supporting information

10.1017/ash.2025.10167.sm001 DiLorenzo et al. supplementary material DiLorenzo et al. supplementary material

## Supplementary material

The supplementary material for this article can be found at https://doi.org/10.1017/ash.2025.10167
