# Institutional readiness to provide critical care to patients with viral hemorrhagic fever (VHF) in the United States after the COVID-19 pandemic – CORRIGENDUM

**DOI:** 10.1017/ash.2026.10302

**Published:** 2026-01-26

**Authors:** Madeline A. DiLorenzo, Anthony LoPiccolo, Wael ElRayes, Jocelyn J. Herstein, Radu Postelnicu, Angela Vasa, Vikramjit Mukherjee

In the initial publication of this article,^1^ author Vikramjit Mukherjee’s name was misspelled. The article has been updated to correct the spelling of the name.
