# Supplementary material for: Institutional readiness to provide critical care to patients with viral hemorrhagic fever (VHF) in the United States after the COVID-19 pandemic
Source: Antimicrob Steward Healthc Epidemiol. 2025 Oct 13;5(1):e257. doi: 10.1017/ash.2025.10167 (PMC12538373; doi:10.1017/ash.2025.10167)
Supplement: DiLorenzo et al. supplementary material [file S2732494X25101678sup001.pdf]

Pre Screen

Please complete the survey below.

Thank you!

Title of Study:Assessing the capacity and capabilities of the United State's special pathogen treatment centers following a once in a century pandemic
s23-00245
Principal Investigator:Vikramjit Mukherjee, MD
Department of Medicine, NYU Grossman School of Medicine
Director, Medical ICU, Bellevue Hospital
462 1st Avenue, New York, NY 10016
Vikramjit.mukherjee@nyulangone.org
703-901-3283

1.About volunteering for this research study

You are being invited to take part in a research study. Your participation is voluntary which means you can choose whether or not you want to take part in this study. Before you can make your decision, you will need to know what the study is about, the possible risks and benefits of being in this study, and what you will have to do in this study. If you have any questions about the study or this form, please ask Anthony Lo Piccolo at (845) 234-3643 or Anthony.LoPiccolo@nyulangone.org or study PI, Virkamjit Mukherjee at Vikramjit.Mukherjee@nyulangone.org.

2.What is the purpose of this study?

The purpose of this study is to assess the capacity and capabilities of state-appointed Special Pathogen Treatment Centers (SPTC) to care for a patient with viral hemorrhagic fever (VHF). The COVID-19 pandemic has put unprecedented strain on United States healthcare facilities. The strain caused by the COVID-19 pandemic may have also catalyzed the development of new policies and investment in new equipment to treat patients with VHFs.

You qualify to take part in this study if you are a critical care staff member working in a state-appointed Special Pathogen Treatment Center (SPTC); or if you work in a facility that has been a SPTC in the past.

3.How long will I be in the study? How many other people will be in the study?

You will be in this study for as long as it takes to complete one survey. Up to 60 people will be in this study.

4.What will I be asked to do in the study?

If you agree to take part in this study, you will be asked to complete a one-time survey about the capabilities and policies at your institution regarding the treatment of VHF patients as well as the impact of the COVID-19 pandemic on them. It will take about 10 minutes of your time. You are free to skip any questions you do not wish to answer.

Information collected and/or used for the purposes of this research will not be used or distributed for future research studies.

5.What are the possible risks or discomforts?

You may feel inconvenienced or mild discomfort from answering survey questions. If that happens, you are free skip or stop answering questions.

6.What are the possible benefits of the study?

You will not benefit from taking part in this study. There is a potential benefit to public health preparedness. As we are emerging from a historic pandemic, it is important to understand the status of the nation's capability and capacity to treat patients with VHF. The information obtained in this study will further our understanding of the nation's preparedness to treat patients with VHF as well as the effect(s) the COVID-19 pandemic may have had on our preparedness.

7.What other choices do I have if I do not participate?

You have the option to not take part in this study. Participation in this study will not affect your relationship with NYU Langone Health.

8.Will I be paid for being in this study?

You will not be paid for taking part in this study.

9.Will I have to pay for anything?

You will not have to pay for anything.

10.When is the study over? Can I leave the Study before it ends?

This survey will be opened for about three months but you only need to complete the survey once and it will take about 10 minutes of your time to complete. Your participation in the study will end as soon as you complete the survey. You may leave the study early by not finishing the survey.

11.How will you protect my confidentiality?

We will protect your confidentiality by making surveys anonymous. PII (Personally Identifiable Information) or PHI (Protected Health Information) or participant contact information will not be collected as part of this survey. Rather, individual participants and their research data will be identified by a unique study identification number generated by RedCap.

What information may be used or shared with others in connection with this study?
•Your anonymous survey responses

Who may use and share information in connection with this study?
The following individuals may use, share, or receive your information for this study:
•The research team, including the Principal Investigator, study coordinators, and personnel responsible for the support or oversight of the study
•Governmental agencies responsible for research oversight

12.The Institutional Review Board (IRB) and how it protects you

The IRB reviews all human research studies - including this study. The IRB follows Federal Government rules and guidelines designed to protect the rights and welfare of the people taking part in the research studies. The IRB also reviews research to make sure the risks for all studies are as small as possible. The NYU Langone Health IRB office number is (212) 263-4110. The NYU Langone Health IRB is made up of doctors, nurses, non-scientists, and people from the community.

13.Who can I call with questions, or if I'm concerned about my rights as a research subject?

If you have questions, concerns or complaints regarding your participation in this research study or if you have any questions about your rights as a research subject, you should speak with the Principal Investigator listed on top of the page 1 of this form. If a member of the research team cannot be reached or you want to talk to someone other than those working on the study, you may contact the NYU Langone Health IRB at (212) 263-4110.

---

1) Is your institution a Special Pathogen Treatment Center recognized by the Administration for Strategic Preparedness and Response (ASPR) or your state department of health?

◯ Yes
◯ No

---

2) Are you currently a member of a clinical team at a Special Pathogen Treatment Center that may take part in treatment of cases of suspected or confirmed VHF?

◯ Yes
◯ No

---

3) What DHHS region of the country are you located in?

◯ Region 1 - CT, MA, ME NH. RI, VT
◯ Region 2 - NY, NJ, PR, VI
◯ Region 3 - DC, DE, MD, PA, VA, WV
◯ Region 4 - AL, FL, GA, KY, MS, NC, SC, TN
◯ Region 5 - IL, IN, MI, MN, OH, WI
◯ Region 6 - AR, LA, NM, OK, TX
◯ Region 7 - IA, KS, MO, NE
◯ Region 8 - CO, MT, ND, SD, UT, WY
◯ Region 9 - AZ, CA, HI, NV, GU, AS, CNMI, RMI, PW
◯ Region 10 - AK, ID, OR, WA

Demographic and Institutional Information

Please complete the survey below.

Thank you!

In what year was your Special Pathogen Treatment Center originally founded? ________________________________

What is your role within the institution?

______

If [institution_role], please designate department: ______ ______ ______ ______ Other department: ______ ______ ______

If Other Health Care Worker, please designate role ________________________________

What is your role within the SPTC?
- ◯ Medical Director
- ◯ Nursing Director
- ◯ Emergency Management
- ◯ Other Clinician
- ◯ Other Administrator

If other clinician, please designate role: ________________________________

If other administrator, please designate role: ________________________________

Was your institution ever a Special Pathogen Treatment Center appointed by the Administration for Strategic Preparedness and Response (ASPR)?______ If yes, what years were you recognized as a Special Pathogen Treatment Center? Start Year- End Year (YYYY-YYYY) ______
If yes, what was your role within the Special Pathogen Treatment Center? ______ If other clinician or administrator, please designate role:______ ______

What is your current role within the institution? ______ If [prior_sptc_inst_role], please designate department: ______ ______ ______ ______ Other department: ______ ______ ______

If Other Health Care Worker, please designate role: ________________________________

Has your unit ever taken care of a patient suspected to have VHF?
- ◯ Yes
- ◯ No

(For this and all subsequent questions, "suspected VHF" refers to a patient under investigation for VHF, but without diagnostic confirmation of VHF.)

Has your unit ever taken care of a patient with confirmed VHF?
- ◯ Yes
- ◯ No

If yes, in what year did the most recent care occur? ________________________________

Does your institution currently have the capacity to care for a patient with suspected or confirmed VHF?
- ◯ Yes
- ◯ No

VHF Policies and Procedures

Please complete the survey below.

Thank you!

Renal Replacement Therapy Policy

Does your institution have a policy governing whether renal replacement therapy should be provided to a patient with suspected or confirmed VHF?

◯ Yes
◯ No

If yes, please summarize/describe the policy below

My unit (select all that apply):

☐ Has provided renal replacement therapy to a suspected VHF
☐ Has provided renal replacement therapy to a confirmed VHF
☐ Is ready to provide renal replacement therapy to a suspected VHF
☐ Is ready to provide renal replacement therapy to a confirmed VHF
☐ Policy is that renal replacement therapy will not be provided to a suspected VHF
☐ Policy is that renal replacement therapy will not be provided to a confirmed VHF

Intubation/ Mechanical Ventilation Policy

Does your institution have a policy governing whether intubation/mechanical ventilation should be provided to a confirmed or suspected VHF?

◯ Yes
◯ No

If yes, please summarize/describe the policy below.

My unit (select all that apply):

☐ Has performed intubation/mechanical ventilation on a suspected VHF
☐ Has performed intubation/mechanical ventilation on a confirmed VHF
☐ Is ready to provide intubation/mechanical ventilation on a suspected VHF
☐ Is ready to provide intubation/mechanical ventilation on a confirmed VHF
☐ Policy is that intubation/mechanical ventilation will not be provided to a suspected VHF
☐ Policy is that intubation/mechanical ventilation will not be provided to a confirmed VHF

Extracorporeal Membrane Oxygenation (ECMO) Policy

Does your institution have a policy governing whether extracorporeal membrane oxygenation (ECMO) should be provided to a confirmed or suspected VHF?

◯ Yes
◯ No

If yes, please summarize/describe the policy below.

My unit (select all that apply):

☐ Has performed extracorporeal membrane oxygenation (ECMO) on a suspected VHF
☐ Has performed extracorporeal membrane oxygenation (ECMO) on a confirmed VHF
☐ Is ready to perform extracorporeal membrane oxygenation (ECMO) on suspected VHF
☐ Is ready to perform extracorporeal membrane oxygenation (ECMO) on a confirmed VHF
☐ Policy is that extracorporeal membrane oxygenation (ECMO) will not be performed on a suspected VHF
☐ Policy is that extracorporeal membrane oxygenation (ECMO) will not be performed on a confirmed VHF

Chest Compression Policy

Does your institution have a policy governing whether chest compressions should be provided to a confirmed or suspected VHF?

◯ Yes
◯ No

If yes, please summarize/describe the policy below.

My unit (select all that apply):

☐ Has performed chest compressions on a suspected VHF
☐ Has performed chest compressions on a confirmed VHF
☐ Is ready to perform chest compressions on a suspected VHF
☐ Is ready to perform chest compressions on a confirmed VHF
☐ Policy is that chest compressions will not be performed on a suspected VHF
☐ Policy is that chest compressions will not be performed on a confirmed VHF

Pharmacological Cardioversion Policy

Does your institution have a policy governing whether pharmacological cardioversion should be provided to a confirmed or suspected VHF?

◯ Yes
◯ No

If yes, please summarize/describe the policy below.

My unit (select all that apply):

☐ Has performed pharmacological cardioversion on a suspected VHF
☐ Has performed pharmacological cardioversion on a confirmed VHF
☐ Is ready to perform pharmacological cardioversion on a suspected VHF
☐ Is ready to perform pharmacological cardioversion on a confirmed VHF
☐ Policy is that pharmacological cardioversion will not be performed on a suspected VHF
☐ Policy is that pharmacological cardioversion will not be performed on a confirmed VHF

Electrical Cardioversion Policy

Does your institution have a policy governing whether electrical cardioversion should be provided to a confirmed or suspected VHF?

◯ Yes
◯ No

If yes, please summarize/describe the policy below.

My unit (select all that apply):

☐ Has performed electrical cardioversion on a suspected VHF
☐ Has performed electrical cardioversion on a confirmed VHF
☐ Is ready to perform electrical cardioversion on a suspected VHF
☐ Is ready to perform electrical cardioversion on a confirmed VHF
☐ Policy is that electrical cardioversion will not be performed on a suspected VHF
☐ Policy is that electrical cardioversion will not be performed on a confirmed VHF

Defibrillation Policy

20.Does your institution have a policy governing whether defibrillation should be provided to a confirmed or suspected VHF?

◯ Yes
◯ No

If yes, please summarize/describe the policy below.

My unit (select all that apply):

☐ Has performed defibrillation on a suspected VHF
☐ Has performed defibrillation on a confirmed VHF
☐ Is ready to perform defibrillation on a suspected VHF
☐ Is ready to perform defibrillation on a confirmed VHF
☐ Policy is that defibrillation will not be performed on a suspected VHF
☐ Policy is that defibrillation will not be performed on a confirmed VHF

Cricothyrotomy Policy

Does your institution have a policy governing whether a cricothyrotomy should be provided to a confirmed or suspected VHF?

◯ Yes
◯ No

If yes, please summarize/describe the policy below.

My unit (select all that apply):

☐ Has performed a cricothyrotomy on a suspected VHF
☐ Has performed a cricothyrotomy on a confirmed VHF
☐ Is ready to perform a cricothyrotomy on a suspected VHF
☐ Is ready to perform a cricothyrotomy on a confirmed VHF
☐ Policy is that cricothyrotomy will not be performed on a suspected VHF
☐ Policy is that cricothyrotomy will not be performed on a confirmed VHF

Code Status Recommendations Policy

Does your institution have a specific policy regarding recommendations for Code Status in a suspected VHF?

◯ Yes
◯ No, we do not have a specific policy regarding Code Status discussions for patients with suspected VHF

If yes, please summarize/describe the policy below.

Does your institution have a specific policy regarding recommendations for Code Status in a confirmed VHF?

◯ Yes
◯ No, we do not have a specific policy regarding Code Status discussions for patients with confirmed VHF

If yes, please summarize/describe the policy below.

On a scale of 1-5 where 1="Does Not Limit Care" and 5="Limits Care", please indicate below how much the following factors impact your institution's decision to provide critical care interventions and cardiopulmonary resuscitation to a suspected VHF:

Staff Safety

◯ 1 ◯ 2 ◯ 3 ◯ 4
◯ 5
(1= Does not Limit Care ---- 5= Limits Care)

Lack of appropriate technology (fitting infection control needs)	◯ 1 ◯ 2 ◯ 3 ◯ 4 ◯ 5 (1= Does not Limit Care ---- 5= Limits Care)
Lack of guidelines on how to provide such care	◯ 1 ◯ 2 ◯ 3 ◯ 4 ◯ 5 (1= Does not Limit Care ---- 5= Limits Care)
Clinical futility	◯ 1 ◯ 2 ◯ 3 ◯ 4 ◯ 5 (1= Does not Limit Care ---- 5= Limits Care)
Limitations of physical building/ward	◯ 1 ◯ 2 ◯ 3 ◯ 4 ◯ 5 (1= Does not Limit Care ---- 5= Limits Care)

On a scale of 1-5 where 1="Does Not Limit Care" and 5="Limits Care", please indicate below how much the following factors impact your institution's decision to provide critical care interventions and cardiopulmonary resuscitation to a confirmed VHF:

Staff Safety	◯ 1 ◯ 2 ◯ 3 ◯ 4 ◯ 5 (1= Does not Limit Care ---- 5= Limits Care)
Lack of appropriate technology (fitting infection control needs)	◯ 1 ◯ 2 ◯ 3 ◯ 4 ◯ 5 (1= Does not Limit Care ---- 5= Limits Care)
Lack of guidelines on how to provide such care	◯ 1 ◯ 2 ◯ 3 ◯ 4 ◯ 5 (1= Does not Limit Care ---- 5= Limits Care)
Clinical futility	◯ 1 ◯ 2 ◯ 3 ◯ 4 ◯ 5 (1= Does not Limit Care ---- 5= Limits Care)
Limitations of physical building/ward	◯ 1 ◯ 2 ◯ 3 ◯ 4 ◯ 5 (1= Does not Limit Care ---- 5= Limits Care)

COVID-19 Pandemic & VHF Preparedness

Please complete the survey below.

Thank you!

Have the strategies (training, staffing or technology) your institution used for the care of a suspected or confirmed VHF changed during the COVID-19 pandemic? Select all that apply.

☐ Yes: Training strategies have changed
☐ Yes: Staffing strategies have changed
☐ Yes: Technologies have changed
☐ No

If yes to any, please summarize/describe below how your institutional strategies for care of a suspected or confirmed VHF have changed during the COVID-19 pandemic. ______________________________

Has your philosophy on resuscitating a suspected VHF changed since the COVID-19 response?

◯ Yes
◯ No

If yes, please summarize/describe how your philosophy on resuscitating a suspected VHF has changed below. ______________________________

Has your philosophy on resuscitating a confirmed VHF changed since the COVID-19 response?

◯ Yes
◯ No

If yes, please summarize/describe how your philosophy on resuscitating a confirmed VHF has changed below. ______________________________

Has the COVID-19 pandemic positively or negatively impacted your facility's ability to care for a suspected or confirmed VHF?

◯ Positively
◯ Negatively
◯ Little to No Difference

Please describe how the COVID-19 pandemic [covid_vhf_care] impacted your facility below: ______________________________

Do you feel that your facility is more or less prepared for a suspected or confirmed VHF patient than in December 2019?

◯ More
◯ Less
◯ Little to No Difference

Please describe how your facility is [covid_vhf_prep] prepared for a suspected or confirmed VHF patient than in December 2019: ______________________________
